# 

**Published:** 1880-01

**Authors:** 


LUCIEN HOWE, M. D.

### CONTENTS:

| | PAGE. |
|---|---|
| **ORIGINAL COMMUNICATIONS:** | |
| Malpractice, by George W. Clinton, L.L.D., | 229 |
| Hemorrhage from the Lungs, | 240 |
| **CLINICAL REPORTS:** | |
| A Case of Puerperal Eclampsia, reported by F. W. Bartlett, M. D., | 248 |
| A Case of Vesicular Mole, reported by A. S. Coe, M. D., | 250 |
| Colloid Degeneration of the Omentum, reported by A. G. Crittenden, M. D., | 254 |
| **TRANSLATIONS:** | |
| The Treatment of Intermittent Convergent Strabismus in Children by Mydriatics or Myotics—By Dr. Boucheron. From the French, by Lucien Howe, M. D., | 255 |
| How to Grasp the Pelvis for Fixation in Contraction of the Hip, by Dr. R. Gersuny. From the German, by Herman Mynter, M. D., | 256 |
| **SELECTIONS:** | |
| Solvents of Gall-Stones, | 257 |
| Phosphide of Zinc, | 259 |
| A New Method of Administering Koosso, | 260 |
| Earache, Chloroform Vapor, | 261 |
| Benzoate of Soda in Diphtheria, | 261 |
| A Simple Method of Preventing Mammary Abscess, | 263 |
| Belladonna in Poisoning by Opium, | 264 |
| Treatment of Colic, | 265 |
| **SOCIETY REPORTS:** | |
| Buffalo Medical Association, | 266 |
| Medical Association of Central New York, | 268 |
| **EDITORIAL:** | |
| Gratuitous Services to Clergymen, | 271 |
| **REVIEWS,** | 272 |

